# MRI-based prognostic factors and risk stratification for treatment response in post-traumatic olfactory dysfunction

**DOI:** 10.3389/fneur.2026.1976254

**Published:** 2026-10-29

**Authors:** Ziyi Wang, Yifan Li, Jixin Luan, Yihan Chen, Shanshan Zhang, Ruoqi Zhang, Tianyang Li, Shijia Wang, Haoyu Yang, Jianfeng Liu

**Affiliations:** 1 Chinese Academy of Medical Sciences & Peking Union Medical College, Beijing, China; 2 Department of Otorhinolaryngology Head and Neck Surgery, China-Japan Friendship Hospital, Beijing, China; 3 Department of Radiology, China-Japan Friendship Hospital, Beijing, China; 4 Graduate School, Beijing University of Chinese Medicine, Beijing, China

**Keywords:** magnetic resonance imaging, olfactory bulb, post-traumatic olfactory dysfunction, prognosis, risk stratification, traumatic brain injury

## Abstract

**Objective:**

To identify baseline magnetic resonance imaging (MRI) features associated with treatment response in post-traumatic olfactory dysfunction (PTOD) and to develop an exploratory MRI-based risk score.

**Methods:**

This single-center retrospective cohort study included 41 patients with PTOD and 41 age- and sex-matched healthy controls. Olfactory bulb (OB) volume, morphology, and integrity, olfactory sulcus depth, and structural lesions in olfactory-related cortical regions were assessed on MRI. Olfactory function was evaluated using the Sniffin’ Sticks test, and treatment response was defined as an increase of ≥5.5 points in the Threshold-Discrimination-Identification score. Least absolute shrinkage and selection operator (LASSO) regression was used to select MRI features for model development, from which a simplified integer-based scoring system was derived.

**Results:**

Compared with healthy controls, patients with PTOD had a shallower left olfactory sulcus and lower proportions of convex OB morphology bilaterally. After a median follow-up of 8 months, 16 of 41 patients (39.0%) met the treatment-response criterion. Three MRI features were retained in the LASSO model: left OB integrity, left orbital gyrus injury, and right temporal lobe injury. The resulting model had an apparent area under the receiver operating characteristic curve (AUC) of 0.885 (95% confidence interval [CI], 0.787–0.983). Full-pipeline internal validation yielded a stratified ten-fold cross-validated AUC of 0.733 (95% CI, 0.564–0.901) and a bootstrap optimism-corrected AUC of 0.725. The simplified three-factor scoring system had an apparent AUC of 0.869 (95% CI, 0.761–0.977) and a bootstrap bias-corrected AUC of 0.867, with treatment response rates of 75.0%, 50.0%, and 5.3% in the low-, intermediate-, and high-risk groups, respectively. The empirical MRI scoring scheme had an apparent AUC of 0.805 (95% CI, 0.662–0.926); the difference between the two scoring systems was not statistically significant (DeLong *p* = 0.079).

**Conclusion:**

Baseline structural MRI features were associated with treatment response in PTOD. The exploratory three-factor MRI-based score showed potential for risk stratification within this cohort, but prospective external validation is required before clinical application.

## 1 Introduction

Post-traumatic olfactory dysfunction (PTOD) is a frequent and clinically important consequence of traumatic brain injury, affecting approximately 7 to 22% of patients following head trauma (1). This condition can substantially diminish quality of life by impairing food enjoyment and nutritional intake, reducing the ability to detect environmental hazards such as gas leaks or smoke, and adversely affecting psychosocial well-being (1, 2). PTOD may result from disruption of olfactory fila at the cribriform plate, direct injury to the olfactory bulbs (OBs), and damage to olfactory-related cortical regions (3, 4). The heterogeneity of these injuries contributes to marked variability in olfactory recovery, making individual prognostic assessment challenging.

Psychophysical testing with the Sniffin’ Sticks provides a standardized assessment of olfactory threshold, discrimination, and identification (5). However, psychophysical testing does not directly characterize the anatomical location or extent of structural injury along the olfactory pathway. Magnetic resonance imaging (MRI) provides complementary structural information, including OB volume and morphology, olfactory sulcus (OS) depth, and structural abnormalities in olfactory-related brain regions (6–8). In PTOD specifically, morphometric MRI studies have demonstrated structural alterations in the OB and in gray- and white-matter regions involved in olfactory processing (8–10).

Beyond structural characterization, several studies have investigated the prognostic relevance of MRI in PTOD. Rombaux et al. found that larger baseline OB volumes were associated with greater subsequent olfactory improvement in patients with postinfectious or post-traumatic olfactory loss (11). Altundag et al. subsequently developed a five-feature imaging-based prognostic scale for predicting response to olfactory training in 52 patients with PTOD (12). More recently, Hsieh et al. showed that MRI lesion patterns across multiple chemosensory-related brain regions were associated with long-term olfactory improvement (13). Collectively, these findings support the prognostic potential of structural MRI, but the optimal combination of imaging features for prognostic stratification in PTOD remains uncertain. Building on these prior approaches, the present study used LASSO-based feature selection to derive a simplified score and directly compared it with an empirical score based on conventional MRI findings.

We hypothesized that a combination of baseline structural MRI features would be associated with treatment response and could be integrated into an exploratory risk score. Therefore, the primary objective was to identify baseline structural MRI features associated with treatment response and use them to derive an exploratory MRI-based risk score. Secondary objectives were to characterize structural differences between patients with PTOD and matched healthy controls and to compare the derived score with an empirical score based on conventional MRI findings.

## 2 Materials and methods

### 2.1 Study design and participants

This single-center retrospective cohort study was conducted at China-Japan Friendship Hospital between January 2018 and September 2025. The study was approved by the Institutional Ethics Review Board of China-Japan Friendship Hospital (Approval No. 2025-KY-362), and the requirement for informed consent was waived because of the retrospective use of anonymized clinical data.

Consecutive patients with PTOD evaluated at our specialized olfactory clinic were screened for eligibility. Inclusion criteria were: (1) a documented history of traumatic brain injury; (2) new-onset persistent olfactory dysfunction or qualitative olfactory disturbance after trauma; (3) a patent olfactory cleft on nasal endoscopy; (4) age ≥18 years; and (5) completion of both MRI and Sniffin’ Sticks testing within 1 week of the initial clinical evaluation, with a Threshold-Discrimination-Identification (TDI) score below the age-adjusted 10th percentile (14). Exclusion criteria were: (1) pre-existing or alternative conditions associated with olfactory dysfunction, including chronic rhinosinusitis, allergic rhinitis, recent upper respiratory tract infection, neurodegenerative disease, or olfactory dysfunction predating the trauma; (2) MRI of insufficient quality for imaging assessment; or (3) missing key clinical or follow-up data.

A total of 41 patients with PTOD met the eligibility criteria. In addition, 41 healthy controls were selected from an institutional healthy-control database and individually matched to patients by sex and age (±5 years). All controls had no history of olfactory dysfunction or relevant neurological disease and underwent complete Sniffin’ Sticks testing to confirm normal olfactory function.

### 2.2 Olfactory function assessment

Olfactory function was assessed using the Sniffin’ Sticks test (Burghart Messtechnik, Wedel, Germany), comprising odor threshold (T), discrimination (D), and identification (I) subtests. The three subtest scores were summed to obtain the composite Threshold-Discrimination-Identification (TDI) score (5). Subjective olfactory function was rated using a visual analog scale (VAS; 0–10). Olfaction-related quality of life was assessed using the Questionnaire of Olfactory Disorders–Negative Statements (QOD-NS), while anxiety and depressive symptoms were evaluated using the Self-Rating Anxiety Scale (SAS) and Self-Rating Depression Scale (SDS), respectively. Treatment response was defined as an increase in TDI score of ≥5.5 points from baseline to follow-up (15).

### 2.3 MRI data acquisition

All subjects underwent high-resolution cranial MRI within 1 week of initial consultation using a 3.0 T MR scanner (Discovery MR750, GE Medical Systems, USA). The standardized protocol included: axial T1-weighted imaging (slice thickness 1.0 mm), axial T2-weighted imaging (slice thickness 5 mm), coronal FLAIR (slice thickness 3 mm), and coronal T2-weighted fast spin-echo sequences optimized for olfactory structure visualization (slice thickness 2.5 mm).

### 2.4 Image analysis

All images were independently evaluated by two readers with neuroradiology training who were blinded to follow-up olfactory outcomes and whether patients were classified as responders or non-responders. Inter-rater reliability was assessed using intraclass correlation coefficients (ICCs) for continuous measurements and Cohen’s κ for categorical assessments. For continuous measurements, the mean of the two readers was used for analysis, whereas categorical disagreements were resolved by consensus.

#### 2.4.1 OB volumetry

Bilateral OB volumes were measured on consecutive coronal T2-weighted images using ITK-SNAP software (version 4.2.0). OB boundaries were manually delineated slice-by-slice according to a previously described manual segmentation method (16). Total volume was calculated as the sum of delineated areas multiplied by slice thickness.

#### 2.4.2 OS depth measurement

OS depth was measured on the coronal plane of the posterior tangent through the eyeballs (PPTE). A line was drawn between the most prominent cortical surfaces of the ipsilateral orbital gyrus (OG) and rectal gyrus (RG), and OS depth was defined as the perpendicular distance from this line to the deepest point of the olfactory sulcus (6, 16).

#### 2.4.3 OB morphology classification

OB morphology was assessed on the coronal T2-weighted image at the PPTE slice and classified according to previously described criteria (7) as convex (olive-shaped, circular, or plano-convex) or non-convex (banana-shaped, irregular, plane, or scattered). Final classification was based on consensus.

#### 2.4.4 OB integrity scoring

A semi-quantitative OB integrity score was developed based on coronal T2-weighted images (Figure 1). For each OB, all consecutive coronal slices on which the OB was visible were evaluated (typically 3–5 slices). A slice was classified as abnormal if any of the following findings was present: signal heterogeneity, malacia, or an unclear contour. The integrity score was calculated as the number of slices showing intact OB structure divided by the total number of evaluated slices. The mean score of the two readers was used for analysis (range, 0–1), with higher scores indicating better OB integrity. For the simplified score, values <1 were classified as impaired to distinguish complete structural integrity from any slice-level structural abnormality; this threshold was not derived by outcome-based optimization.

**Figure 1:**
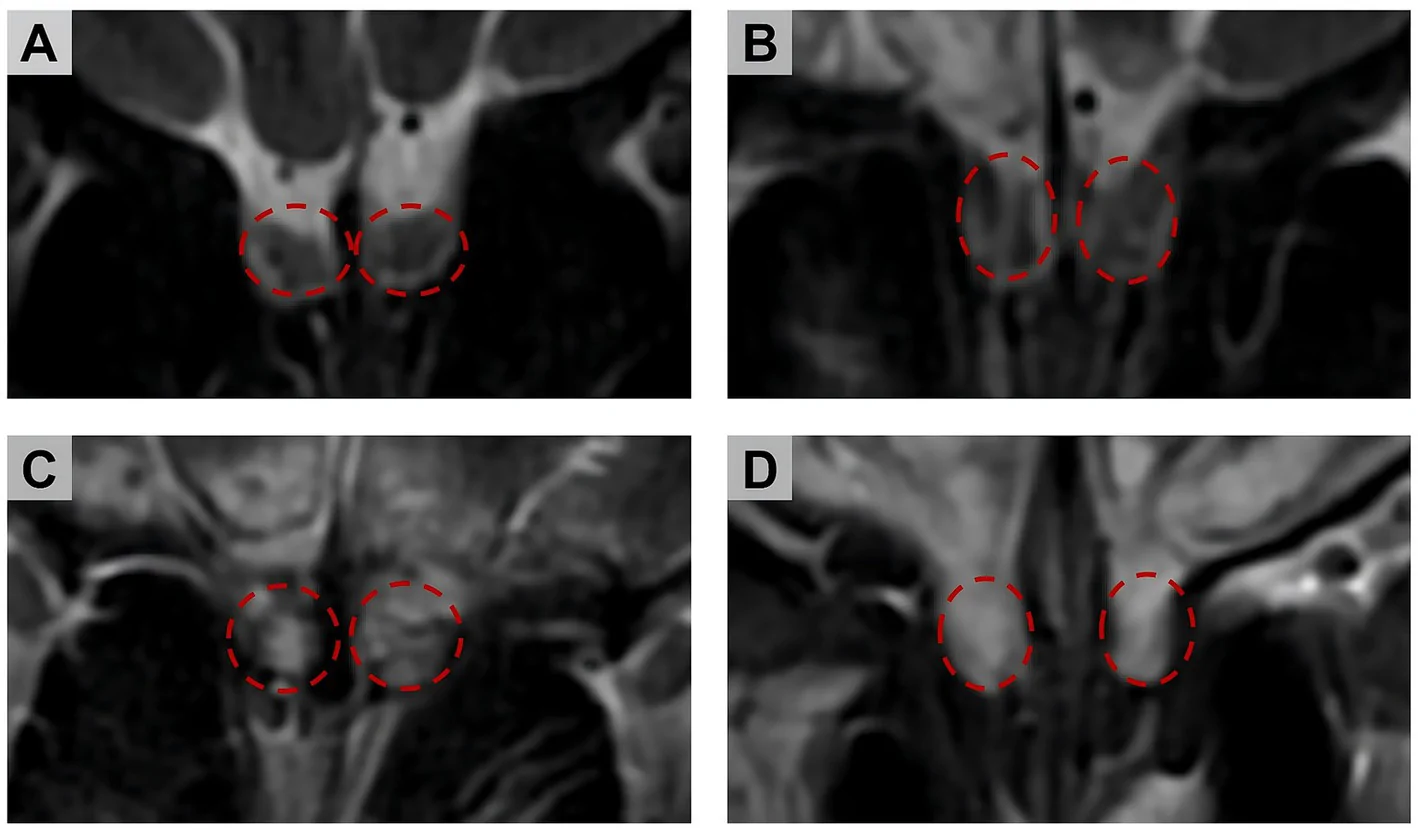
Representative OB imaging findings in patients with PTOD (coronal T2WI). **(A)** Normal olfactory bulb: clear, symmetrical contour, homogeneous signal. **(B)** Heterogeneous OB signal: focal increased or mixed signal intensity within the OB. **(C)** Malacia: obvious malacic focus within the OB. **(D)** Unclear OB contour: blurred boundaries between the OB and surrounding CSF/brain parenchyma, with discontinuous margins on some slices, making accurate delineation difficult.

#### 2.4.5 Assessment of cortical lesions

Structural lesions in the orbital gyri, rectal gyri, and temporal lobes were systematically evaluated separately for each hemisphere on conventional MRI sequences. Anatomical localization was determined according to standard neuroanatomical landmarks on multiplanar MRI. Lesions were recorded as present or absent based on focal abnormalities including encephalomalacia, hemosiderin deposition, or gliosis.

#### 2.4.6 Empirical scoring scheme construction

To explore a simple scoring approach suitable for routine MRI interpretation, an empirical scoring scheme was constructed based on visual assessment of conventional MRI. Seven imaging features were evaluated bilaterally: unclear OB boundary, OB malacia, heterogeneous OB signal, OG injury, RG injury, temporal lobe injury, and injury to other brain regions, yielding 14 side-specific items. Each abnormal finding was assigned 1 point (normal = 0), resulting in a total score ranging from 0 to 14.

### 2.5 Treatment and follow-up

All patients were prescribed standardized olfactory training consisting of nine odorants: clove, acetic acid, peppermint, rosemary, citronella, jasmine, orange, eucalyptus, and an unpleasant odor. Patients were instructed to smell each odor for 10 s, with a 10-s interval between odors, sequentially through all nine odorants, three times daily, for at least 3 months. They were advised to avoid smoking and alcohol for 1 h before training.

Adjunctive therapies, including mecobalamin, *Ginkgo biloba* extract, mouse nerve growth factor, prednisolone acetate, budesonide, and zinc sulfate, were prescribed at the discretion of the treating physician according to individual clinical conditions. Adjunctive treatment use was recorded as a binary variable (use vs. non-use) based on the available medical records. Exact treatment duration and adherence to olfactory training were not systematically documented. MRI findings were not used to guide treatment decisions.

Olfactory function was reassessed during follow-up using the Sniffin’ Sticks test. When multiple follow-up olfactory assessments were available, the latest available Sniffin’ Sticks assessment was used to define treatment response. Treatment response was determined according to the change in TDI score from baseline to follow-up.

### 2.6 Statistical analysis

Statistical analyses were performed using SPSS version 26.0 and R version 4.5.2. Continuous variables were assessed for normality using the Shapiro–Wilk test and are presented as mean ± standard deviation or median (interquartile range), as appropriate. Between-group comparisons were performed using independent-samples *t* tests, Welch’s *t* tests, or Mann–Whitney U tests, as appropriate. Categorical variables were compared using the Pearson chi-square test or Fisher’s exact test. Effect sizes were reported with 95% confidence intervals (CIs), and Spearman correlation analysis was used to assess associations between subjective and objective olfactory measures. For exploratory phase-based analyses, patients with PTOD were categorized according to the interval from trauma to baseline assessment as acute (≤3 months) or chronic (>3 months).

Eleven candidate variables selected on the basis of univariate *p* < 0.20 or clinical relevance were entered into least absolute shrinkage and selection operator (LASSO) logistic regression. Ten-fold cross-validation with the λ.1se criterion was used to favor a more parsimonious model. Apparent model performance in the development cohort was assessed by the area under the receiver operating characteristic curve (AUC), calibration slope, and Brier score. Additional full-pipeline internal validation was performed using nested cross-validation and bootstrap resampling. In the outer stratified 10-fold cross-validation, predictor screening was repeated within each training fold, followed by LASSO tuning using stratified five-fold cross-validation. The complete model-development procedure was also repeated within 1,000 bootstrap samples for optimism correction. These estimates were considered measures of internal validation only and were not interpreted as evidence of external generalizability. Bootstrap bias correction was applied separately to the AUC estimates of the simplified and empirical scoring systems. As a sensitivity analysis, multivariable logistic regression including the three-factor MRI score and follow-up duration was performed to assess whether the association between the MRI-based score and treatment response persisted after accounting for follow-up duration.

A simplified integer-based score was derived from the LASSO-selected predictors, with higher scores indicating greater risk of nonresponse. Patients were categorized as low risk (0–2 points), intermediate risk (3 points), or high risk (4–5 points), and response rates were compared using Fisher’s exact test. The empirical MRI scoring scheme was evaluated similarly. For the empirical scoring scheme, *post hoc* pairwise comparisons between risk groups were performed using Fisher’s exact tests with Bonferroni correction. AUCs of the two scoring systems were compared using the paired DeLong test. Agreement between their three-level risk classifications was assessed using the exact agreement rate and linear weighted Cohen’s κ, with the 95% CI for κ estimated using 1,000 bootstrap resamples. All tests were two-sided, with *p* < 0.05 considered statistically significant. This was an exploratory study, and no *a priori* sample size calculation was performed.

## 3 Results

### 3.1 Baseline characteristics

A total of 41 patients with PTOD and 41 age- and sex-matched healthy controls were included. The median age was 37 years (IQR, 27–45) in the PTOD group and 36 years (IQR, 26–45) in the control group. Women accounted for 58.5% of participants in both groups.

Among patients with PTOD, the median interval from trauma to the initial olfactory assessment was 13 weeks (IQR, 9–29.5). Traffic accidents were the most common mechanism of injury (61.0%), followed by falls (31.7%). The occipital region was the most common primary impact site (75.6%), and 46.3% of patients had a history of unconsciousness. Phantosmia and parosmia were reported by 48.8 and 17.1% of patients, respectively.

At baseline, patients with PTOD had a median TDI score of 7 (IQR, 6–9) and a median VAS score of 0 (IQR, 0–1). The median QOD-NS and SAS scores were 26 (IQR, 16–29) and 42.5 (IQR, 38.13–48.75), respectively, whereas the mean SDS score was 53.75 ± 7.96. Detailed baseline characteristics are presented in Table 1.

**Table 1:** Baseline characteristics of PTOD patients (*n* = 41).

Characteristic	PTOD Group (*n* = 41)
Demographics	
Age [years, M (IQR)]	37 (27–45)
Sex, female [*n* (%)]	24 (58.5)
Trauma characteristics	
Time from trauma to assessment [weeks, M (IQR)]	13 (9–29.5)
Main trauma mechanism [*n* (%)]	
Traffic accident	25 (61.0)
Fall	13 (31.7)
Other	3 (7.3)
Primary impact site [*n* (%)]	
Occipital	31 (75.6)
Other	10 (24.4)
History of unconsciousness [*n* (%)]	19 (46.3)
Baseline olfactory function	
TDI score [M (IQR)]	7 (6–9)
Threshold (T)	0.5 (0–1)
Discrimination (D)	4 (3–5)
Identification (I)	3 (2.5–4)
VAS score [M (IQR)]	0 (0–1)

PTOD, post-traumatic olfactory dysfunction; M, median; IQR, interquartile range; TDI, Threshold-Discrimination-Identification; VAS, Visual Analog Scale.

### 3.2 Correlation between subjective and objective olfactory measures

Spearman correlation analysis showed significant positive correlations between baseline VAS scores and all objective olfactory measures, with the strongest correlation observed for the total TDI score (Spearman’s ρ = 0.594, *p* < 0.001).

### 3.3 Comparison of MRI findings between PTOD patients and healthy controls

Inter-rater reliability was excellent for all imaging assessments, with ICCs and Cohen’s κ values exceeding 0.80.

#### 3.3.1 Olfactory bulb morphology

OB morphology could be reliably classified in 37 of the 41 patients with PTOD; the remaining four patients were excluded from the morphology analysis because the OB shape could not be reliably determined. Convex OB morphology was less frequent in patients with PTOD than in healthy controls in both hemispheres (left: 51.4% vs. 78.0%; RD = −26.6%, 95% CI, −46.5% to −6.7%; *p* = 0.010; right: 56.8% vs. 78.0%; RD = −21.2%, 95% CI, −41.0% to −1.4%; *p* = 0.044) (Figures 2A,B).

**Figure 2:**
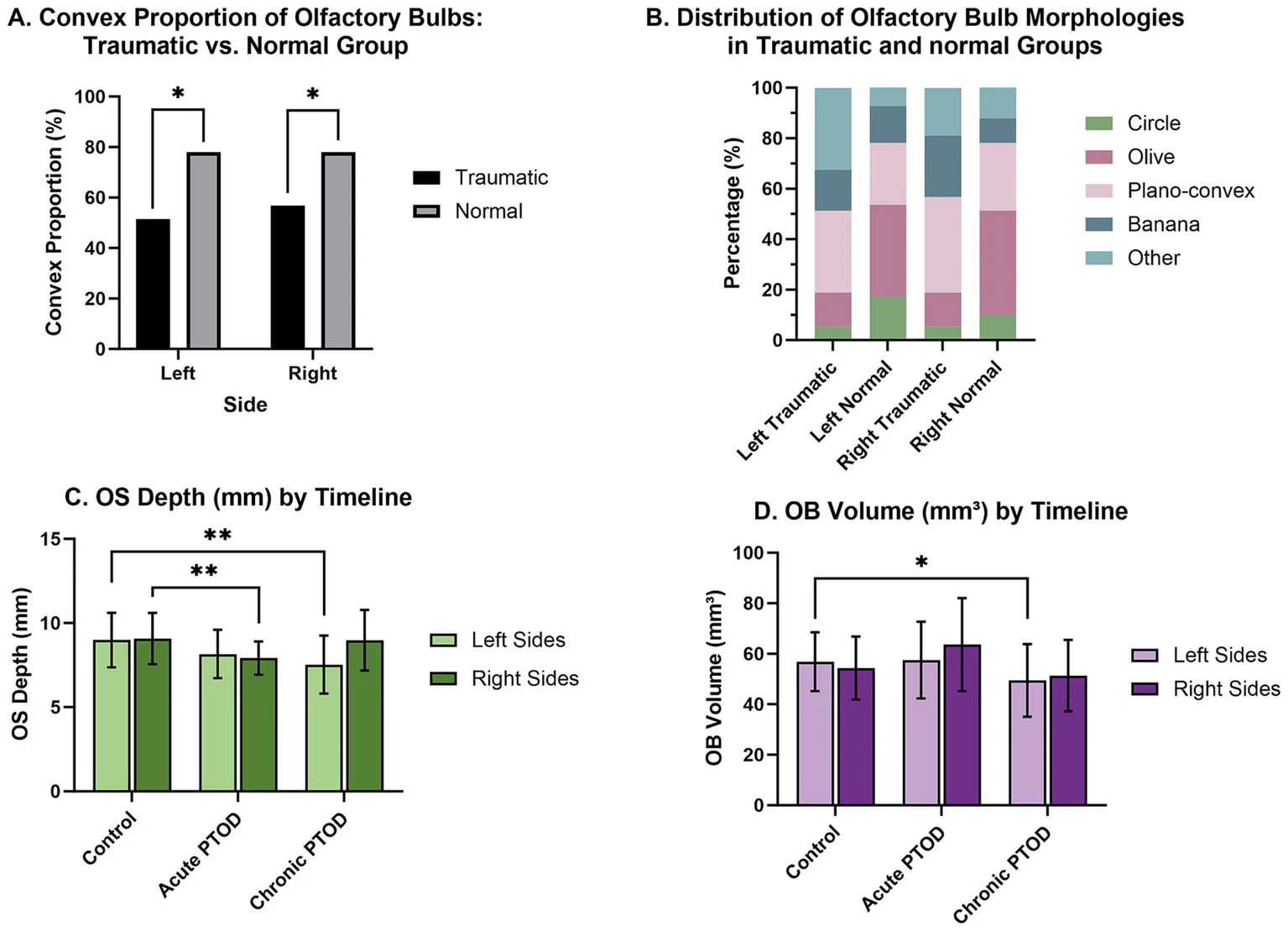
MRI structural characteristics of the olfactory pathway in patients with PTOD. **(A)** Comparison of bilateral convex OB morphology between patients with PTOD and healthy controls. The PTOD group showed significantly lower proportions of convex OB morphology in both hemispheres. **(B)** Stacked bar chart showing the distribution of OB morphological types. The “Other” category includes irregular, plane, and scattered shapes. **(C)** Exploratory phase-related differences in olfactory sulcus (OS) depth: significant right-sided OS shallowing was observed in the acute phase (≤3 months post-trauma, *p* = 0.005), whereas significant left-sided OS shallowing was observed in the chronic phase (>3 months, *p* = 0.007). **(D)** Exploratory phase-related differences in olfactory bulb (OB) volume: right OB volume was numerically larger in the acute phase (*p* = 0.056), whereas left OB volume was significantly lower in the chronic phase (*p* = 0.042). *p < 0.05; **p < 0.01.

#### 3.3.2 Olfactory sulcus depth

Left OS depth was significantly shallower in patients with PTOD than in healthy controls (7.91 ± 1.56 vs. 8.99 ± 1.62 mm; mean difference = −1.08 mm, 95% CI, −1.82 to −0.34; *p* = 0.005). Right OS depth was also shallower in patients with PTOD, although the difference did not reach statistical significance (8.38 ± 1.48 vs. 9.07 ± 1.52 mm; mean difference = −0.69 mm, 95% CI, −1.40 to 0.02; *p* = 0.055). In exploratory subgroup analyses, significant right-sided OS shallowing was observed in the acute phase (≤3 months; *p* = 0.005), whereas significant left-sided OS shallowing was observed in the chronic phase (>3 months; *p* = 0.007) (Figure 2C).

#### 3.3.3 Olfactory bulb volume

In the acute phase, right OB volume did not differ significantly between patients with PTOD and healthy controls (63.63 ± 18.45 vs. 54.33 ± 12.46 mm^3^; mean difference = 9.30 mm^3^, 95% CI, −0.28 to 18.88; *p* = 0.056). In the chronic phase, left OB volume was significantly lower in patients with PTOD than in healthy controls (49.44 ± 14.38 vs. 56.83 ± 11.65 mm^3^; mean difference = −7.38 mm^3^, 95% CI, −14.48 to −0.29; *p* = 0.042) (Figure 2D).

### 3.4 Development and evaluation of the prognostic model for treatment response

#### 3.4.1 Treatment response

After a median follow-up of 8 months (range, 3–75 months), the median TDI score increased from 7 (IQR, 6–9) at baseline to 9 (IQR, 7–18) at follow-up (*p* < 0.001). Follow-up duration did not differ significantly between responders and non-responders (median, 8.0 months [IQR, 5.75–16.25] vs. 8.0 months [IQR, 4.00–14.00], respectively; *p* = 0.437). Overall, 16 of 41 patients (39.0%) met the predefined criterion for treatment response. Among responders, the median post-treatment TDI score was 18.5 (IQR, 15.5–25.0), with a median increase of 11 points; 62.5% of responders improved from anosmia to hyposmia.

#### 3.4.2 Univariate analysis

Univariate analysis identified several imaging variables associated with treatment response (full results in Supplementary Table S1). Responders showed higher left OB integrity (median difference = 0.50, *p* = 0.003) and lower rates of left OB unclear margin, left RG injury, left OG injury, and bilateral temporal lobe injury (all *p* < 0.05).

Parosmia was numerically more frequent in responders (*p* = 0.132). No demographic or trauma-related variables, including history of unconsciousness and its duration, skull fracture, and intracranial hematoma, were significantly associated with treatment response. Likewise, the use of mecobalamin, *Ginkgo biloba* extract, mouse nerve growth factor, prednisolone acetate, budesonide, or zinc sulfate did not differ significantly between responders and non-responders (all *p* > 0.05; Supplementary Table S2).

#### 3.4.3 LASSO regression and variable selection

Eleven candidate variables were entered into the LASSO logistic regression based on the predefined selection criteria. Ten-fold cross-validation using the λ.1se criterion (λ.1se = 0.114) retained three features: left OB integrity (β = 0.42), left OG injury (β = −1.27), and right temporal lobe injury (β = −0.47) (Table 2; Figure 3A).

**Table 2:** LASSO-derived three-factor prognostic model.

MRI feature	*β*	Score
Left OB integrity	0.42	1 (if integrity score < 1)
Left OG injury	−1.27	3
Right temporal lobe injury	−0.47	1

Predictors were selected using LASSO logistic regression with the 1-SE criterion (λ.1se = 0.114). The β coefficients were oriented toward treatment response, whereas the simplified score was oriented toward risk of nonresponse. Left OB integrity was modeled as a continuous variable (range, 0–1; higher values indicate better integrity); for the simplified score, an integrity score <1 was considered impaired and assigned 1 point. LASSO, least absolute shrinkage and selection operator; OB, olfactory bulb; OG, orbital gyrus.

**Figure 3:**
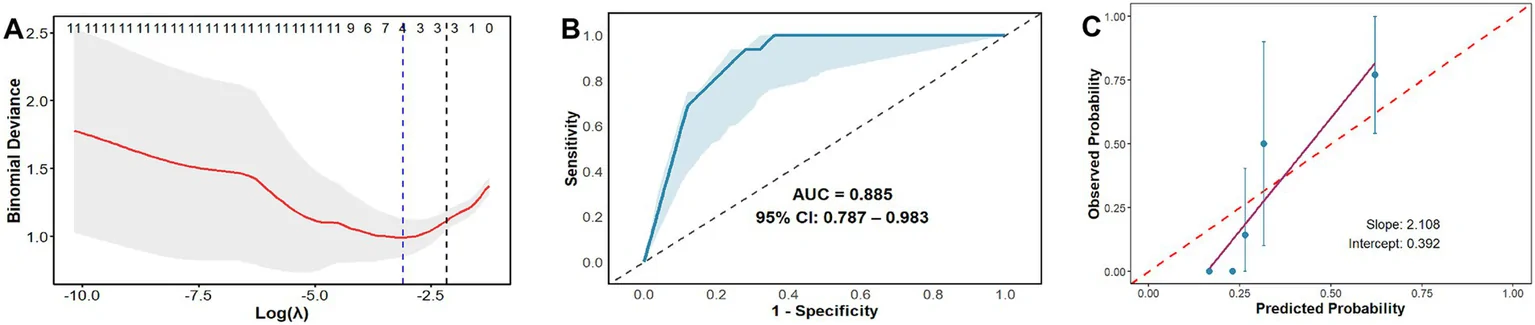
Least absolute shrinkage and selection operator (LASSO) variable selection and apparent performance of the three-factor prognostic model. **(A)** Ten-fold cross-validation curve for LASSO logistic regression. The x-axis represents log(λ), and the y-axis represents binomial deviance. The curve shows the mean cross-validated deviance, with error bars representing standard errors. Vertical dashed lines indicate λ.min and λ.1se; three predictors were retained at λ.1se = 0.114. **(B)** Receiver operating characteristic curve of the LASSO-derived three-factor model (apparent AUC = 0.885, 95% CI, 0.787–0.983). The diagonal dashed line represents no discrimination. **(C)** Apparent calibration plot comparing predicted and observed probabilities. The diagonal dashed line represents perfect calibration. Points represent observed response probabilities across grouped predicted-probability intervals, with vertical bars representing 95% confidence intervals. Calibration slope = 2.108, intercept = 0.392, and Brier score = 0.156.

The resulting three-factor model had an apparent AUC of 0.885 (95% CI, 0.787–0.983) (Figure 3B). The apparent calibration slope was 2.11 and the Brier score was 0.156 (Figure 3C). In the additional full-pipeline internal validation, the stratified 10-fold cross-validated AUC was 0.733 (95% CI, 0.564–0.901), and full-pipeline bootstrap validation with 1,000 resamples yielded a consistent optimism-corrected AUC of 0.725. To facilitate interpretation, the model was subsequently simplified into an integer-based scoring system.

#### 3.4.4 Development of the three-factor scoring system

The three selected predictors were converted into a simplified integer-based risk score by dividing the absolute value of each LASSO coefficient by the smallest absolute coefficient and rounding the resulting ratios to the nearest integer. Impaired left OB integrity (integrity score < 1) was assigned 1 point, left OG injury 3 points, and right temporal lobe injury 1 point, yielding a total score ranging from 0 to 5. Patients were stratified into low-risk (0–2 points, *n* = 16), intermediate-risk (3 points, *n* = 6), and high-risk (4–5 points, *n* = 19) groups. Treatment response rates decreased progressively across the three groups, from 75.0% (12/16; 95% CI, 50.5–89.8%) to 50.0% (3/6; 95% CI, 18.8–81.2%) and 5.3% (1/19; 95% CI, 0.9–24.6%), respectively (Fisher’s exact *p* < 0.001) (Table 3). The scoring system had an apparent AUC of 0.869 (95% CI, 0.761–0.977). The bootstrap bias-corrected AUC was 0.867 (95% CI, 0.776–0.985). In a sensitivity analysis adjusting for follow-up duration, the three-factor MRI score remained significantly associated with treatment response (OR per 1-point increase, 0.36; 95% CI, 0.21–0.63; *p* < 0.001), whereas follow-up duration was not significantly associated with treatment response (OR per month, 1.04; 95% CI, 0.99–1.09; *p* = 0.105).

**Table 3:** Risk stratification and treatment response rates for the three-factor scoring system.

Risk group	Score range	*n*	Responders *n* (%)	95% CI for response rate (%)
Low-risk	0–2 points	16	12 (75.0)	50.5–89.8
Intermediate-risk	3 points	6	3 (50.0)	18.8–81.2
High-risk	4–5 points	19	1 (5.3)	0.9–24.6

The three-factor score assigned 1 point for impaired left OB integrity (integrity score < 1), 3 points for left OG injury, and 1 point for right temporal lobe injury. Differences in treatment response across the three risk groups were assessed using Fisher’s exact test (p < 0.001). Confidence intervals for response rates were calculated using the Wilson method. OB, olfactory bulb; OG, orbital gyrus; CI, confidence interval.

#### 3.4.5 Performance of the empirical scoring scheme

The empirical scoring scheme stratified patients into low-risk (≤7 points, *n* = 17), intermediate-risk (8–9 points, *n* = 11), and high-risk (≥10 points, *n* = 13) groups, with treatment response rates of 64.7% (11/17), 36.4% (4/11), and 7.7% (1/13), respectively. Treatment response rates differed significantly across the three groups (Fisher’s exact *p* = 0.006). In pairwise comparisons with Bonferroni correction, the difference between the low- and high-risk groups remained significant (*p* = 0.002), whereas comparisons involving the intermediate-risk group were not significant. The empirical scoring scheme had an apparent AUC of 0.805 (95% CI, 0.662–0.926). Bootstrap assessment yielded a bias-corrected AUC of 0.804 (95% CI, 0.652–0.933) (Figure 4).

**Figure 4:**
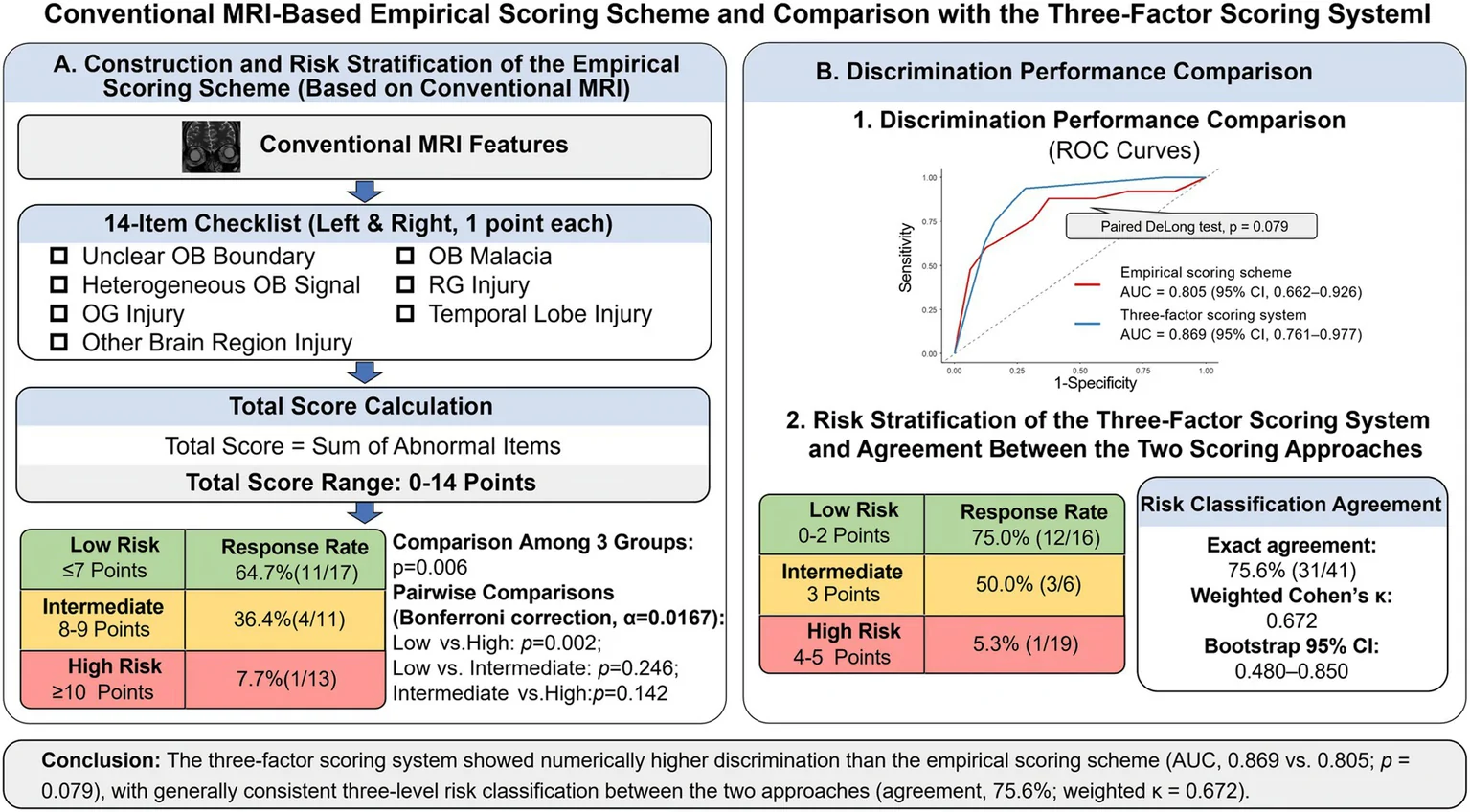
Conventional MRI-based empirical scoring scheme and comparison with the three-factor scoring system. **(A)** Construction and risk stratification of the empirical scoring scheme based on conventional MRI. Seven imaging features were evaluated bilaterally, yielding 14 side-specific items, with 1 point assigned to each abnormal finding and a total score ranging from 0 to 14. Patients were classified as low risk (≤7 points), intermediate risk (8–9 points), or high risk (≥10 points), with treatment response rates of 64.7% (11/17), 36.4% (4/11), and 7.7% (1/13), respectively (Fisher’s exact *p* = 0.006). **(B)** Comparison of discrimination and risk stratification between the empirical and three-factor scoring systems. The empirical scoring scheme had an apparent AUC of 0.805 (95% CI, 0.662–0.926), compared with an apparent AUC of 0.869 (95% CI, 0.761–0.977) for the three-factor scoring system; the difference was not statistically significant (paired DeLong *p* = 0.079). In the three-factor scoring system, treatment response rates were 75.0% (12/16), 50.0% (3/6), and 5.3% (1/19) in the low-risk (0–2 points), intermediate-risk (3 points), and high-risk (4–5 points) groups, respectively. The two scoring systems showed 75.6% exact agreement (31/41) in three-level risk classification, with a linear weighted Cohen’s κ of 0.672 (bootstrap 95% CI, 0.480–0.850).

#### 3.4.6 Comparison of prognostic scoring systems

The three-factor scoring system had a numerically higher apparent AUC than the empirical scoring scheme (0.869 vs. 0.805), although the difference in discrimination did not reach statistical significance in the paired DeLong test (*p* = 0.079). The two scoring systems showed an exact agreement of 75.6% (31/41) in three-level risk classification, with a linear weighted Cohen’s κ of 0.672 (bootstrap 95% CI, 0.480–0.850).

## 4 Discussion

### 4.1 Principal findings

This retrospective cohort study systematically characterized structural MRI features of the olfactory pathway in patients with PTOD and identified baseline imaging features associated with treatment response. Three main findings emerged. First, patients with PTOD exhibited structural differences compared with healthy controls, including shallower left OS depth and a lower prevalence of convex OB morphology bilaterally, with exploratory phase-related differences in OS depth and OB volume. Second, LASSO regression retained three MRI features—left OB integrity, left OG injury, and right temporal lobe injury—and the resulting three-factor model demonstrated discrimination within the present cohort. The simplified scoring system derived from these features further stratified patients into groups with progressively lower treatment response rates. Third, the empirical MRI scoring scheme also showed discriminatory ability, although its apparent AUC was numerically lower than that of the three-factor scoring system. The two scoring systems showed 75.6% exact agreement in three-level risk classification, with a weighted Cohen’s κ of 0.672.

### 4.2 Structural alterations in PTOD patients

The relationship between OS depth and olfactory function is complex and generally modest. A large-scale study involving more than 1,000 participants showed that the association between OS depth and olfactory function was attenuated after adjustment for age, suggesting a substantial influence of age on this measure (17). Studies in patients with olfactory loss have likewise reported weak or absent correlations between OS depth and odor identification or TDI scores, with only a modest association between right OS depth and odor threshold (18). Against this background, the shallower left OS observed in our age-matched PTOD cohort may reflect trauma-related structural alterations rather than age-related variation. The lower prevalence of convex OB morphology is also consistent with previous work linking OB shape to olfactory function (7). Previous studies have reported reduced OB volume in PTOD and associations between OB volume and olfactory impairment (8, 19, 20). In our cohort, the acute and chronic subgroups showed different patterns of OS depth and OB volume, including numerically larger right OB volume in the acute phase and significantly lower left OB volume in the chronic phase. These exploratory cross-sectional findings may reflect temporal differences in post-traumatic structural changes, but longitudinal imaging is required to characterize their evolution.

### 4.3 Prognostic factors for treatment response

The three predictors—left OB integrity, left OG injury, and right temporal lobe injury—represent structural features at different levels of the olfactory pathway that may be relevant to treatment response. Given the observational design, these associations should not be interpreted as evidence of causal mechanisms.

Preserved left OB integrity was positively associated with treatment response. As the first central relay for olfactory information, structural preservation of the OB may support transmission of regenerating olfactory inputs and neural plasticity during olfactory rehabilitation. This finding is consistent with previous studies linking OB structural measures, particularly OB volume, to olfactory function and recovery (11, 19, 21). More broadly, preserved structural integrity along the olfactory pathway may provide a substrate for rehabilitation-related neural plasticity rather than serving solely as a static prognostic marker. Previous neuroimaging studies in patients with post-traumatic olfactory dysfunction have reported structural and functional changes following olfactory training, including alterations in cortical morphology and resting-state effective connectivity within olfactory-related regions (22, 23). Emerging neuromodulatory approaches, including repetitive transcranial magnetic stimulation, may provide another means of engaging residual olfactory networks and promoting functional plasticity, although their therapeutic role in PTOD remains preliminary (24). However, whether baseline structural preservation determines the capacity for such plasticity remains uncertain.

Left OG injury was associated with a lower likelihood of treatment response. The orbitofrontal region is an important component of higher-order olfactory networks and shows strong functional connectivity with primary olfactory cortical regions (25). Structural damage in this region may therefore interfere with the integration of olfactory information required for functional improvement.

Right temporal lobe injury was also associated with nonresponse. Hsieh et al. (13) reported that MRI lesion patterns involving multiple chemosensory-related brain regions were associated with long-term olfactory improvement in PTOD, supporting the relevance of central structural injury to prognosis. Functional imaging studies have implicated medial temporal regions, particularly the amygdala and parahippocampal region, in olfactory hedonic processing and odor-evoked autobiographical memory (26, 27); disruption of these networks may therefore be relevant to recovery.

The asymmetric lateralization of the selected predictors—left-sided OB and OG involvement together with right-sided temporal injury—should be interpreted cautiously. Given the small sample size, this side-specific pattern may reflect the distribution of lesions within the present cohort. Any mechanistic interpretation remains speculative, and the present data cannot determine whether the observed lateralization reflects true hemispheric specialization or cohort-specific injury patterns.

### 4.4 Exploratory risk stratification and potential implications

The simplified three-factor scoring system provides an exploratory framework for risk stratification in PTOD. Treatment response rates decreased from 75.0% in the low-risk group to 50.0% in the intermediate-risk group and 5.3% in the high-risk group. However, these estimates should be interpreted cautiously given the modest sample size, particularly the small number of patients in the intermediate-risk group (*n* = 6). Accordingly, the proposed risk categories should be regarded as exploratory and require external validation before clinical use.

The empirical scoring scheme also showed discriminatory ability and was based entirely on conventional MRI findings without specialized image-processing software. The three-factor scoring system had a numerically higher apparent AUC than the empirical scoring scheme (0.869 vs. 0.805), although the difference was not statistically significant. The two scoring systems showed 75.6% exact agreement in three-level risk classification, with a weighted Cohen’s κ of 0.672, suggesting broadly similar, although not identical, patient stratification. Together, these findings support further evaluation of routine structural MRI for exploratory prognostic stratification in PTOD.

### 4.5 Limitations and future directions

Several limitations should be acknowledged. First, the retrospective single-center design and limited number of responders (16 of 41 patients) relative to the 11 candidate predictors may limit model stability and generalizability. Selection bias may also result from recruitment at a specialized olfactory clinic and the requirement for complete imaging, olfactory testing, and follow-up data. Although penalized LASSO regression and full-pipeline internal validation were used to reduce overfitting and assess model optimism, external validation in larger prospective cohorts is still required before clinical application. Second, adjunctive therapies were not standardized, and treatment duration and adherence to olfactory training were not systematically documented, which may have introduced residual confounding. In addition, spontaneous recovery after trauma may have contributed to the observed improvement in olfactory function. Third, the simplified scoring system required dichotomization of the semi-quantitative left OB integrity measure, and the proposed risk-group cutoffs were derived from the present cohort; therefore, these categories should be regarded as exploratory. Fourth, only structural MRI was analyzed, and the observed lateralization of predictors may reflect cohort-specific injury patterns rather than true hemispheric specialization.

Future studies should prioritize prospective multicenter validation, longitudinal imaging of olfactory pathway changes, and integration of advanced imaging modalities to further refine MRI-based prognostic assessment.

## 5 Conclusion

Left OB integrity, left OG injury, and right temporal lobe injury were associated with treatment response in patients with PTOD. An exploratory three-factor MRI-based scoring system derived within this small single-center cohort stratified patients into groups with differing treatment response rates, while the empirical MRI scoring scheme also demonstrated discriminatory ability. These hypothesis-generating findings support further investigation of structural MRI for prognostic stratification in PTOD, but prospective multicenter external validation is required before clinical application.

## Data Availability

The datasets generated and analyzed during the current study are not publicly available due to patient privacy considerations but are available from the corresponding author upon reasonable request.

## References

[1] Patel ZM Holbrook EH Turner JH Adappa ND Albers MW Altundag A . International consensus statement on allergy and rhinology: olfaction. Int Forum Allergy Rhinol. (2022) 12:327–680. doi: 10.1002/alr.22929, 35373533 PMC12261282

[2] Ahmedy F Mazlan M Danaee M Abu Bakar MZ. Post-traumatic brain injury olfactory dysfunction: factors influencing quality of life. Eur Arch Otorhinolaryngol. (2020) 277:1343–51. doi: 10.1007/s00405-020-05823-0, 32025786

[3] Howell J Costanzo RM Reiter ER. Head trauma and olfactory function. World J Otorhinolaryngol Head Neck Surg. (2018) 4:39–45. doi: 10.1016/j.wjorl.2018.02.001, 30035260 PMC6051255

[4] Gao X Wu D Li X Su B Sun Z Nie B . Altered glucose metabolism of the olfactory-related cortices in anosmia patients with traumatic brain injury. Eur Arch Otorhinolaryngol. (2021) 278:4813–21. doi: 10.1007/s00405-021-06754-0, 33744988

[5] Hummel T Sekinger B Wolf SR Pauli E Kobal G. Sniffin' sticks': olfactory performance assessed by the combined testing of odor identification, odor discrimination and olfactory threshold. Chem Senses. (1997) 22:39–52. doi: 10.1093/chemse/22.1.39, 9056084

[6] Rombaux P Potier H Markessis E Duprez T Hummel T. Olfactory bulb volume and depth of olfactory sulcus in patients with idiopathic olfactory loss. Eur Arch Otorhinolaryngol. (2010) 267:1551–6. doi: 10.1007/s00405-010-1230-2, 20300763

[7] Yan X Joshi A Zang Y Assunção F Fernandes HM Hummel T. The shape of the olfactory bulb predicts olfactory function. Brain Sci. (2022) 12:128. doi: 10.3390/brainsci12020128, 35203892 PMC8870545

[8] Han P Winkler N Hummel C Hähner A Gerber J Hummel T. Alterations of brain gray matter density and olfactory bulb volume in patients with olfactory loss after traumatic brain injury. J Neurotrauma. (2018) 35:2632–40. doi: 10.1089/neu.2017.5393, 29699465

[9] Rezaeyan A Asadi S Kamrava SK Zare-Sadeghi A. Brain structural analysis in patients with post-traumatic anosmia: voxel-based and surface-based morphometry. J Neuroradiol. (2023) 50:482–91. doi: 10.1016/j.neurad.2022.11.005, 36610937

[10] Gao X Su B Sun Z Xu L Wei Y Wu D. Patterns of gray and white matter volume alterations in patients with post-traumatic anosmia: a voxel-based morphometry study. Front Neurol. (2022) 13:690760. doi: 10.3389/fneur.2022.690760, 35860485 PMC9289146

[11] Rombaux P Huart C Deggouj N Duprez T Hummel T. Prognostic value of olfactory bulb volume measurement for recovery in postinfectious and posttraumatic olfactory loss. Otolaryngol Head Neck Surg. (2012) 147:1136–41. doi: 10.1177/0194599812459704, 22951433

[12] Altundag A Saatci O Kandemirli SG Sanli DET Duz OA Sanli AN . Imaging features to predict response to olfactory training in post-traumatic olfactory dysfunction. Laryngoscope. (2021) 131:E2243–E2250. doi: 10.1002/lary.29392, 33449371

[13] Hsieh JW Lenoir V Sipione R Hugentobler M Daskalou D Lundstrom JN . Can MRI predict olfactory loss and improvement in posttraumatic olfactory dysfunction? Rhinology. (2024) 62:172–82. doi: 10.4193/Rhin23.246, 37955246

[14] Oleszkiewicz A Schriever VA Croy I Hähner A Hummel T. Updated Sniffin' sticks normative data based on an extended sample of 9139 subjects. Eur Arch Otorhinolaryngol. (2019) 276:719–28. doi: 10.1007/s00405-018-5248-1, 30554358 PMC6411676

[15] Gudziol V Lötsch J Hähner A Zahnert T Hummel T. Clinical significance of results from olfactory testing. Laryngoscope. (2006) 116:1858–63. doi: 10.1097/01.mlg.0000234915.51189.cb, 17003712

[16] Duprez TP Rombaux P. Imaging the olfactory tract (cranial nerve #1). Eur J Radiol. (2010) 74:288–98. doi: 10.1016/j.ejrad.2009.05.065, 20303227

[17] Li Z Abdul Manan H Heitmann H Witte V Wirkner K Riedel-Heller S . The association between depth of the olfactory sulcus, age, gender and olfactory function: an MRI-based investigation in more than 1000 participants. Neuroscience. (2023) 519:31–7. doi: 10.1016/j.neuroscience.2023.03.017, 36934780

[18] Hummel T Urbig A Huart C Duprez T Rombaux P. Volume of olfactory bulb and depth of olfactory sulcus in 378 consecutive patients with olfactory loss. J Neurol. (2015) 262:1046–51. doi: 10.1007/s00415-015-7691-x, 25712545

[19] Rombaux P Mouraux A Bertrand B Nicolas G Duprez T Hummel T. Retronasal and orthonasal olfactory function in relation to olfactory bulb volume in patients with posttraumatic loss of smell. Laryngoscope. (2006) 116:901–5. doi: 10.1097/01.mlg.0000217533.60311.e7, 16735894

[20] Mueller A Rodewald A Reden J Gerber J von Kummer R Hummel T. Reduced olfactory bulb volume in post-traumatic and post-infectious olfactory dysfunction. Neuroreport. (2005) 16:475–8. doi: 10.1097/00001756-200504040-00011, 15770154

[21] Buschhüter D Smitka M Puschmann S Gerber JC Witt M Abolmaali ND . Correlation between olfactory bulb volume and olfactory function. NeuroImage. (2008) 42:498–502. doi: 10.1016/j.neuroimage.2008.05.004, 18555701

[22] Rezaeyan A Asadi S Kamrava SK Khoei S Zare-Sadeghi A. Reorganizing brain structure through olfactory training in post-traumatic smell impairment: an MRI study. J Neuroradiol. (2022) 49:333–42. doi: 10.1016/j.neurad.2021.04.035, 33957160

[23] Hosseini K Zare-Sadeghi A Sadigh-Eteghad S Mirsalehi M Khezerloo D. Effects of olfactory training on resting-state effective connectivity in patients with posttraumatic olfactory dysfunction. Acta Neurobiol Exp (Wars). (2020) 80:381–8. doi: 10.21307/ane-2020-035, 33350991

[24] Di Fazio C Palermo S. A prefrontal Neuromodulation route for post-traumatic olfactory dysfunction: a perspective supported by recovery during left-DLPFC rTMS. Brain Sci. (2026) 16:99. doi: 10.3390/brainsci16010099, 41594820 PMC12839034

[25] Zhou G Lane G Cooper SL Kahnt T Zelano C. Characterizing functional pathways of the human olfactory system. eLife. (2019) 8:e47177. doi: 10.7554/eLife.47177, 31339489 PMC6656430

[26] Zou LQ van Hartevelt TJ Kringelbach ML Cheung EFC Chan RCK. The neural mechanism of hedonic processing and judgment of pleasant odors: an activation likelihood estimation meta-analysis. Neuropsychology. (2016) 30:970–9. doi: 10.1037/neu0000292, 27195988

[27] Arshamian A Iannilli E Gerber JC Willander J Persson J Seo HS . The functional neuroanatomy of odor evoked autobiographical memories cued by odors and words. Neuropsychologia. (2013) 51:123–31. doi: 10.1016/j.neuropsychologia.2012.10.023, 23147501

