# Supplementary material for: MRI-based prognostic factors and risk stratification for treatment response in post-traumatic olfactory dysfunction
Source: Front Neurol. 2026 Oct 29;17:1976254. doi: 10.3389/fneur.2026.1976254 (PMC13754747; doi:10.3389/fneur.2026.1976254)
Supplement: Supplementary file 1 [file Table_1.doc]

SUPPLEMENT

Table S1. Univariate Analysis of All Baseline Variables Between Treatment Responders and Non-Responders

| Variable | Responders (n=16) | Non-responders (n=25) | Effect size (95% CI) | P-value |
| --- | --- | --- | --- | --- |
| Demographics |  |  |  |  |
| Age [years, M(IQR)] | 37 (30–42) | 35.5 (27–48.75) | 0.0 (−8.0 to 8.0) | 0.98 |
| Sex, male [n (%)] | 6 (37.5) | 11 (44.0) | −6.5% (−37.2% to 24.2%) | 0.68 |
| Trauma Characteristics |  |  |  |  |
| Trauma mechanism [n (%)] |  |  | – | 0.43 |
| Traffic accident | 9 (56.3) | 16 (64.0) |  |  |
| Assault | 0 (0.0) | 2 (8.0) |  |  |
| Fall | 6 (37.5) | 7 (28.0) |  |  |
| Fall from height | 1 (6.3) | 0 (0.0) |  |  |
| Primary impact site [n (%)] |  |  | – | 0.34 |
| Occipital | 12 (75.0) | 19 (76.0) |  |  |
| Facial | 3 (18.8) | 2 (8.0) |  |  |
| Temporal | 0 (0.0) | 3 (12.0) |  |  |
| Parietal | 1 (6.3) | 0 (0.0) |  |  |
| Frontal | 0 (0.0) | 1 (4.0) |  |  |
| Time to assessment [weeks, M(IQR)] | 17.00 (9.00–31.50) | 12.00 (5.25–23.50) | 4.5 (−3.0 to 17.0) | 0.22 |
| History of unconsciousness [n (%)] | 8 (50.0) | 11 (44.0) | 6.0% (−25.3% to 37.3%) | 0.71 |
| Duration of unconsciousness [n (%)] |  |  | – | 0.46 |
| No unconsciousness | 8 (50.0) | 14 (56.0) |  |  |
| < 1 hour | 5 (31.3) | 4 (16.0) |  |  |
| 1–24 hours | 3 (18.8) | 4 (16.0) |  |  |
| > 24 hours | 0 (0.0) | 3 (12.0) |  |  |
| Skull fracture [n (%)] | 11 (68.8) | 15 (60.0) | 8.8% (−20.4% to 38.0%) | 0.57 |
| Intracranial hematoma [n (%)] | 8 (50.0) | 15 (60.0) | −10.0% (−40.5% to 20.5%) | 0.53 |
| Clinical Symptoms |  |  |  |  |
| Phantosmia [n (%)] | 8 (50.0) | 12 (48.0) | 2.0% (−29.0% to 33.0%) | 0.90 |
| Parosmia [n (%)] | 5 (31.3) | 2 (8.0) | 23.3% (−2.0% to 48.6%) | 0.13*‡ |
| Baseline Olfactory Function |  |  |  |  |
| Threshold (T) [M(IQR)] | 1 (0–1) | 0 (0–1) | 0.0 (−1.0 to 0.0) | 0.11* |
| Discrimination (D) [M(IQR)] | 3.5 (3–5) | 4 (3–5) | 0.0 (−1.0 to 0.0) | 0.57 |
| Identification (I) [M(IQR)] | 3 (3–5) | 3 (2–3.5) | 0.0 (−1.0 to 0.0) | 0.30 |
| TDI total score [M(IQR)] | 7.5 (6–9.75) | 7 (6.25–8.25) | 0.0 (−2.0 to 1.0) | 0.67 |
| VAS score [M(IQR)] | 0 (0–1) | 0 (0–1) | 0.0 (0.0 to 0.0) | 0.42 |
| Taste-Related Symptoms |  |  |  |  |
| Hypogeusia [n (%)] | 5 (31.3) | 11 (44.0) | −12.7% (−42.8% to 17.4%) | 0.41 |
| Parageusia [n (%)] | 1 (6.3) | 0 (0.0) | 6.3% (−5.6% to 18.2%) | 0.39 |
| Phantogeusia [n (%)] | 2 (12.5) | 1 (4.0) | 8.5% (−8.4% to 25.4%) | 0.69 |
| Imaging Features |  |  |  |  |
| Left OB integrity [M(IQR)] | 1.00 (1.00–1.00) | 0.50 (0.00–1.00) | 0.50 (0.00 to 0.75) | **0.003** *‡ |
| Right OB integrity [M(IQR)] | 1.00 (0.56–1.00) | 0.75 (0.13–1.00) | 0.00 (−0.50 to 0.00) | 0.23 ‡ |
| Left unclear margin [n (%)] | 3 (18.8) | 14 (56.0) | −37.2% (−64.5% to −9.9%) | **0.018** *‡ |
| Right unclear margin [n (%)] | 6 (37.5) | 14 (56.0) | −18.5% (−48.8% to 11.8%) | 0.25 ‡ |
| Left OB malacia [n (%)] | 11 (68.8) | 22 (88.0) | −19.2% (−43.8% to 5.4%) | 0.27 |
| Right OB malacia [n (%)] | 11 (68.8) | 22 (88.0) | −19.2% (−43.8% to 5.4%) | 0.27 |
| Left OB signal heterogeneity [n (%)] | 14 (87.5) | 23 (92.0) | −4.5% (−21.9% to 12.9%) | >0.99 |
| Right OB signal heterogeneity [n (%)] | 15 (93.8) | 23 (92.0) | 1.8% (−11.8% to 15.4%) | >0.99 |
| Left OB volume [mm³, Mean±SD] | 52.37 ± 13.93 | 54.52 ± 16.19 | −2.16 (−12.49 to 8.18) | 0.68 |
| Right OB volume [mm³, Mean±SD] | 53.80 ± 14.86 | 61.15 ± 19.03 | −7.35 (−19.43 to 4.73) | 0.23 |
| Left OB convex shape [n (%)] | 9 (60.0) | 10 (45.5) | 14.5% (−16.5% to 45.5%) | 0.51 |
| Right OB convex shape [n (%)] | 7 (46.7) | 14 (63.6) | −16.9% (−47.8% to 14.0%) | 0.34 |
| Left OS depth [mm, Mean±SD] | 8.14 ± 1.69 | 7.69 ± 1.44 | 0.46 (−0.66 to 1.57) | 0.41 |
| Right OS depth [mm, Mean±SD] | 8.39 ± 1.44 | 8.38 ± 1.55 | 0.01 (−1.08 to 1.10) | 0.98 |
| Left RG injury [n (%)] | 8 (50.0) | 20 (80.0) | −30.0% (−57.0% to −3.0%) | **0.044** *‡ |
| Right RG injury [n (%)] | 9 (56.3) | 19 (76.0) | −19.7% (−48.5% to 9.1%) | 0.19 *‡ |
| Left OG injury [n (%)] | 4 (25.0) | 21 (84.0) | −59.0% (−80.0% to −38.0%) | **<0.001** *‡ |
| Right OG injury [n (%)] | 7 (43.8) | 18 (72.0) | −28.2% (−57.5% to 1.1%) | 0.07 *‡ |
| Left temporal lobe injury [n (%)] | 0 (0.0) | 8 (32.0) | −32.0% (−50.3% to −13.7%) | **0.034** *‡ |
| Right temporal lobe injury [n (%)] | 0 (0.0) | 8 (32.0) | −32.0% (−50.3% to −13.7%) | **0.034** *‡ |
| Left other regions injury [n (%)] | 0 (0.0) | 2 (8.0) | −8.0% (−20.6% to 4.6%) | 0.51 |
| Right other regions injury [n (%)] | 0 (0.0) | 2 (8.0) | −8.0% (−20.6% to 4.6%) | 0.51 |

Note: Data are presented as median (interquartile range), mean ± standard deviation, or frequency (percentage). Continuous variables were compared using the Mann–Whitney U test or independent samples t-test, as appropriate. Categorical variables were compared using the Pearson chi-square test, continuity-corrected chi-square test, or Fisher’s exact test. Effect sizes are presented as median differences (Hodges–Lehmann estimator) for non-normally distributed variables, mean differences for normally distributed variables, and risk differences (RDs) for categorical variables, all with 95% confidence intervals (CIs).* Variables with p < 0.20 were considered for inclusion in LASSO regression. ‡ Candidate variables entered into LASSO regression. Bold P-values indicate statistical significance at α = 0.05

Table S2. Association between adjunctive treatment modalities and treatment response

| Treatment | Responders, n (%) | Non-responders, n (%) | OR (95% CI) | P-value |
| --- | --- | --- | --- | --- |
| Mecobalamin | 7 (43.8) | 17 (68.0) | 0.37 (0.10–1.34) | 0.195 |
| Ginkgo biloba extract | 15 (93.8) | 24 (96.0) | 0.63 (0.04–10.76) | 1.000 |
| Mouse nerve growth factor | 15 (93.8) | 19 (76.0) | 4.74 (0.51–43.73) | 0.215 |
| Prednisolone acetate | 6 (37.5) | 10 (40.0) | 0.90 (0.25–3.27) | 1.000 |
| Budesonide | 13 (81.3) | 23 (92.0) | 0.38 (0.06–2.56) | 0.362 |
| Zinc sulfate | 7 (43.8) | 9 (36.0) | 1.38 (0.38–4.98) | 0.746 |

Note: Odds ratios (ORs) were calculated for treatment use versus non-use. P-values were calculated using the chi-square test or Fisher’s exact test
